# Unleashing the Potential: Exploring the Application and Mechanism of Mesenchymal Stem Cells in Autoimmune Diseases

**DOI:** 10.1155/sci/9440377

**Published:** 2025-04-15

**Authors:** Xinqi Li, Rongli Li, Jialing Huang, Yuelin Hu, Chenxi Fan, Xin Wang, Hongsong Yu

**Affiliations:** ^1^Department of Immunology, Special Key Laboratory of Gene Detection and Therapy of Guizhou Province, Zunyi Medical University, Zunyi, China; ^2^School of Basic Medical Sciences, Special Key Laboratory of Ocular Diseases of Guizhou Province, Zunyi Medical University, Zunyi, China

**Keywords:** applications, autoimmune disease, clinical trials, mechanisms, mesenchymal stem cells

## Abstract

Autoimmune diseases (AIDs) occur when the immune system mistakenly attacks the body's own antigens. Traditionally, these conditions are treated with nonspecific immunosuppressive therapies, including corticosteroids, immunosuppressants, biological agents, and human immunoglobulins. However, these treatments often fail to achieve optimal outcomes, especially for patients with severe cases. Mesenchymal stem cells (MSCs) present a promising alternative due to their robust self-renewal capabilities and multidirectional differentiation potential. MSCs are easily accessible, exhibit low immunogenicity, and can help reduce graft rejection. MSCs can inhibit T cell proliferation, reduce proinflammatory T cells, inhibit B cell differentiation, induce macrophage polarization towards the anti-inflammatory M2 phenotype, and suppress activity of natural killer (NK) cells and dendritic cells (DCs). Additionally, MSCs can regulate T cells, macrophages, and fibroblast-like synoviocytes (FLS) by releasing microRNA (miRNA) through exosomes or extracellular vesicles (EVs), thus providing therapeutic benefits for various diseases. Numerous clinical trials have highlighted the therapeutic benefits of MSCs in treating various AIDs, leading to increased interest in MSC transplantation. This review summarizes the current applications and mechanisms of action of MSCs in the treatment of AIDs.

## 1. Introduction

Autoimmune diseases (AIDs) occur when the immune system mistakenly attacks the body, resulting from a failure in the self-tolerance mechanism. This failure leads to the overactivity of immune cells, the generation of autoantibodies, and the release of numerous inflammatory factors [1]. AIDs can be classified into organ-specific or systemic types. Organ-specific diseases, such as multiple sclerosis (MS), affect only a particular organ. In contrast, systemic diseases, like rheumatoid arthritis (RA) and systemic lupus erythematosus (SLE), impact the entire body. The development of AIDs is multifaceted and complex, with potential triggers including environmental influences, genetic predisposition, and infections [2]. Fundamentally, these diseases arise from a breakdown of self-immune tolerance. Imbalances between host defensive responses and inflammatory reactions, caused by disrupted complement function, cytokine imbalances, and excessive activation of immune cells, contribute to the onset of AIDs [3].

Genetic susceptibility also plays a significant role in AIDs. Individuals carrying specific genetic variants, especially in major histocompatibility complex (MHC) molecules, are more likely to develop these diseases [4]. Abnormal expression of any MHC class can trigger AIDs. Human leukocyte antigens (HLA), encoded by MHC genes, are strongly correlated with AID development. Different HLA subtypes have variations in their amino acid sequences responsible for antigen presentation. Consequently, individuals with distinct HLA subtypes vary in their ability to present exogenous and endogenous peptides, leading to differences in their susceptibility to AIDs [5].

Currently, glucocorticoids or traditional disease-modifying antirheumatic drugs (DMARDs) remain the preferred initial treatment for patients with AIDs. While these treatments are effective for most patients, long-term use can lead to various adverse effects. Additionally, the sensitivity of the affected organs to different immunosuppressive agents can widely vary. Although biological agents have shown remarkable efficacy, they carry a high risk of allergies and severe infections, and their cost is a significant barrier. Given these challenges, there is an urgent need for new therapeutic approaches. Mesenchymal stem cell (MSC) therapy, an emerging treatment, holds significant promise for the treatment of various AIDs and could be a beacon of hope for patients.

MSCs generally refer to mesenchymal stem cells or mesenchymal stromal cells. According to the definition of the International Society for Cell Therapy (ISCT), MSCs are defined as adult stem cells possessing adhesive, multipotent, and self-renewing properties, capable of differentiating into various adult cell types. Mesenchymal stromal cells, on the other hand, are a cultured in vitro cell group with multipotency and tissue regeneration support but lacking strict stem cell characteristics, commonly utilized in clinical and laboratory settings for tissue repair and therapy. Since our article covers both *in vitro* and in vivo mechanisms as well as cellular therapy, we will refrain from reiterating the concepts as mentioned [6].

MSCs can be derived from various sources, including bone marrow, adipose tissue, and the umbilical cord. MSCs from different sources vary in their immunophenotype, proliferation rate, differentiation ability, and gene expression. Despite these differences, they share many biological features such as a high capacity for self-renewal, multidirectional differentiation potential, and robust tissue repair capabilities. They can also inhibit mitosis-induced T cell proliferation in vitro, evade immune surveillance in vivo, and participate in immune regulation [7, 8]. Additionally, MSCs can inhibit the differentiation and proliferation of effector T cells, B cells, natural killer (NK) cells, and dendritic cells (DCs) [9]. Furthermore, they can regulate the immune response during chronic inflammation by managing the recruitment and function of cells in both the innate and adaptive immune systems [10, 11]. Therefore, MSCs could potentially reduce the likelihood of recipient rejection after transplantation and mitigate the severity of rejection [2–4, 12]. To date, most studies have found that MSCs exert therapeutic effects on AIDs primarily through two pathways: paracrine signaling and direct cell-to-cell contact [5, 13].

Recent studies have shown that the dysregulation of microRNA (miRNA) in patients with AIDs plays a significant role in disease progression [14]. The therapeutic effects of MSCs on AIDs may be mediated by their secretion of miRNAs into the extracellular space through various cell types, forming lipid or lipoprotein complexes such as extracellular vesicles (EVs) and exosomes. When these EVs and exosomes interact with target cells, they can alter the function of those cells [15]. The biological impact of exosomes on recipient cells is largely influenced by the specific miRNAs they carry. Research has identified certain miRNAs abundant in MSC-derived exosomes that exhibit unique immunomodulatory properties [16]. However, the molecular mechanisms through which MSCs regulate circulating miRNAs remain largely unclear. Overall, these characteristics underscore the potential of MSCs as a therapeutic approach in clinical applications and research related to AIDs.

SLE and RA are common AIDs that have attracted substantial research interest, leading to significant progress in understanding their pathogenesis, diagnosis, and treatment. Between 2020 and 2023, more than 8000 articles on SLE and over 27,000 on RA were published in PubMed. Type 1 diabetes (T1DM), MS, and immune thrombocytopenia (ITP) are prevalent organ-specific AIDs. Extensive research also exists on T1DM, MS, and ITP. Similarly, analysis of U.S. data from commercially insured individuals demonstrated an overall lower incidence in individuals 20–64 years of age (18.6/100,000/year) than in youth aged 0–19 years (34.3/100,000/year), but the total number of new cases in adults over a 14-year period was 19,174 compared with 13,302 in youth [17]. From 2016 to 2018, a Chinese study screened a total of 27,336 hospitalizations of 15,060 patients in 1665 tertiary hospitals, of which 9879 were new cases. The age- and sex-adjusted incidence of MS was 0.235 per 100,000 per year, 0.055 per 100,000 in children, and 0.288 per 100,000 in adults [18]. The annual incidence of ITP is estimated to be 2–5/100,000, and the incidence rate of women of childbearing age is higher than that of men of the same age group, and the sex tends to be the same for those over 60 years old [19]. This review explores the therapeutic mechanisms underlying MSCs in the treatment of five AIDs: SLE, RA, T1DM, MS, and ITP. It also addresses the current challenges associated with the use of MSCs for treating these conditions.

## 2. MSCs and AIDs

### 2.1. MSCs and SLE

SLE is an autoimmune disorder that affects multiple organs and systems and is characterized by the presence of autoantibodies in the serum. The disease is most prevalent in females aged 15–40 and is more common in children than adults. The prognosis depends on the type and extent of organ involvement [20]. The development of SLE is linked to the abnormal activation of T and B lymphocytes, which leads to the production of large numbers of abnormal autoantibodies and the deposition of these cells in organs and tissues, causing damage [21].

Research indicates that, in addition to abnormal immune cell activation, SLE is also characterized by defects in hematopoietic stem cells and MSCs [22]. For patients with severe or refractory disease, stem cell transplantation is the preferred treatment method. Consolidation therapy for SLE may also involve low-dose oral glucocorticoids, biologics, or immunosuppressive agents [23]. Studies have shown a high rate of SLE recurrence after allogeneic MSC transplantation alone. To reduce recurrence and achieve sustained disease remission, combining allogeneic MSC transplantation with autologous hematopoietic stem cell transplantation is recommended [24, 25].

#### 2.1.1. Regulation of T Cells by MSCs in SLE

In SLE patients, T cell immunity is disrupted, resulting in increased T cell expression. Those with active disease exhibit an imbalance between T helper cell 17 (Th17) and regulatory T cell (treg) numbers, marked by an increase in Th17 cells and a decrease in Tregs. These dysregulated immune cell activities contribute to the pathogenesis of SLE. MSCs produce various immunoregulatory and growth factors that modulate T cell immune responses [26]. For example, MSCs secrete paracrine factors like TGF-*β*1 and PGE2, which promote Treg production and inhibit Th1/Th17 responses [27]. The immunosuppressive effects of bone marrow-derived MSCs (BMSCs) on CD8^+^ T lymphocytes involve interactions between the NKG2D receptor on CD8^+^ T lymphocytes and MHC Class I chain-related (MIC) proteins A and B ligands on BMSC surfaces. This downregulates NKG2D expression on CD8^+^ T lymphocytes, inhibiting their proliferation and functions, thus reducing the cytotoxic capacity mediated by cytotoxic T lymphocytes (CTLs) against alloantigens [28].

Additionally, MSCs can inhibit the differentiation of Th0 cells into Th17 cells, as well as Th1 cells, while promoting their differentiation into Th2 cells. Th2 cells secrete anti-inflammatory cytokines such as interleukin-10 (IL-10), which inhibits SLE-associated inflammation [29]. Huang et al. [30] concluded that treatment with human UC-MSCs (hUC-MSCs) significantly alleviated both functional and renal histopathological damage in mice with SLE, mediated through TGF-*β*1 signaling. Both in vitro and in vivo experiments showed that hUC-MSC treatment protected glomerular podocytes.

Adipose-derived MSCs (ADMSCs) injected through the tail vein into SLE mice resulted in increased proportions of DCs and Tregs and transformed Th cell subtypes, thereby affecting the immune function of SLE mice [31]. Therefore, MSC transplantation helps maintain the balance of cell subsets, promotes immune tolerance, and delays the progression of SLE.

#### 2.1.2. Regulation of B Cells by MSCs in SLE

In SLE, there is abnormal B cell proliferation, leading to increased numbers of memory B cells and significant production of autoantibodies. MSCs induce stagnation in the B-cell growth cycle, reducing plasma cell generation and immunoglobulin levels [32]. They regulate B cells by inhibiting B lymphocyte proliferation and differentiation, thereby reducing B cell activation and antibody production. The inhibition of follicular and marginal B lymphocyte proliferation and differentiation into plasma cells by BMSCs depends on cytokines like interferon-*γ* (IFN-*γ*). MSCs also produce chemokine ligand 2 (CCL2), a potent inflammatory chemotactic factor for monocytes and macrophages, which prevents immunoglobulin production by plasma cells. However, CCL2 alone does not inhibit B lymphocyte proliferation and differentiation; it requires the presence of both CCL2 and matrix metalloproteinases [33]. A key molecular mechanism involves the interaction between MSCs and the inhibitory molecule programed death-1 (PD-1) and its ligand PD-L1. The PD-L1 on MSCs and PD-1 on B lymphocytes interaction inhibits autoreactive B cell activation. MSCs also downregulate the expression of clusters of differentiation 27 (CD27) and 28 (CD28), inhibiting IgM and IgG secretion [33]. These findings demonstrate that MSCs exert an immunoregulatory effect on abnormally activated B cells in SLE patients.

#### 2.1.3. Regulation of NK Cells by MSCs in SLE

In SLE patients, NK cells exhibit reduced expression of CD3zeta, transforming them into a proinflammatory phenotype with altered functions. Exosomes are small vesicles secreted by most cells, characterized by a lipid bilayer structure, with diameters ranging from 40–200 nm [34]. MSCs, in particular, produce high levels of exosomes, which contain various cytokines, growth factors, metabolic products, and miRNAs [35]. When exosomes fuze with target cell membranes, they release their contents, inducing regulatory signals in the recipient cells [36]. Both MSCs and their exosomes inhibit NK cell proliferation, differentiation, and maturation by suppressing surface marker expression on NK cells, inhibiting the proliferation and activation of resting NK cells, and ultimately reducing NK cell cytotoxicity [37].

#### 2.1.4. Regulation of DCs by MSCs in SLE

DCs are the most efficient antigen-presenting cells (APCs), capable of acquiring, processing, transporting, and expressing a wide range of antigens. Numerous studies have shown that MSCs exert an immunosuppressive effect on DCs. Treatment of DCs with MSC-derived exosomes decreases IL-4 expression while increasing TGF-*β* expression, which stimulates Treg differentiation and plays an immunosuppressive role. MSCs also inhibit IL-12 secretion from DCs [36]. MSCs co-cultivated with peripheral blood mononuclear cells (PBMCs) from SLE patients significantly reduced CD11c expression in DCs, suppressed proinflammatory factors such as IFN-*γ* and IL-6, and increased the anti-inflammatory factor IL-10 secretion. Additionally, MSCs downregulated DC maturation markers and inhibited tumor necrosis factor-*α* (TNF-*α*) secretion, suggesting that MSCs regulate the proinflammatory environment of PBMCs in SLE patients by inhibiting DC function [38].

#### 2.1.5. Regulation of Macrophages by MSCs in SLE

Macrophages are divided into two subtypes: M1 macrophages, which have proinflammatory effects, and M2 macrophages, which express cytokines related to type 2 immune responses and anti-inflammatory processes. Studies show that M1 macrophages play a significant proinflammatory role in SLE pathogenesis [39]. SLE is characterized by abnormal macrophage apoptosis, dysfunctional antigen presentation, and activation of autoreactive B cells, leading to increased autoantibody production and tissue damage [40]. MSCs promote the differentiation of macrophages into the M2 phenotype and inhibit M1 differentiation, enhancing pathogen clearance and killing by macrophages. High levels of M2 macrophages are beneficial for alleviating inflammation and thus the symptoms of SLE patients. Studies have shown that both local and systemic applications of MSC-exosomes (MSC-exos) effectively inhibit tissue inflammatory responses, promoting the survival and regeneration of damaged parenchymal cells [41].

#### 2.1.6. Regulation of RNA by MSCs and MSC-Exos in SLE

Circulating miRNAs are small, single-stranded noncoding RNAs found in various body fluids that play a crucial role in regulating immune and inflammatory pathways by inhibiting mRNA translation or promoting mRNA degradation [42]. Research has shown that the expression of miR-320b and its target gene MAP3K1 is closely associated with the activity of SLE. After MSC transplantation, levels of circulating miR-320b and MAP3K1 significantly decreased, which helped alleviate SLE symptoms and inhibited CD4^+^ T cell proliferation in MRL/lpr mice [15]. Additionally, studies indicate that exosomes secreted during MSC transplantation transfer Fas to recipient MRL/lpr bone marrow stem cells (BMSCs), leading to a reduction in intracellular miR-29b levels. This process restores DNA methyltransferase 1 (Dnmt1)-mediated hypomethylation of the Notch1 promoter, thereby enhancing the function of MRL/lpr BMSCs. Overall, these findings reveal how MSC transplantation can improve MRL/lpr BMSC function through the exosome-mediated regulation of the miR-29b/Dnmt1/Notch epigenetic cascade [43]. Furthermore, small RNA sequencing has identified key tRNA-derived fragments (tRFs) in MSC-exos and their effects on macrophage polarization. Notably, TSNA-21109 was found to be upregulated in response to MSC-exos treatment, suggesting that MSC-exos may inhibit M1-type polarization in macrophages by transferring TSNA-21109, which could reduce inflammation and alleviate symptoms in SLE patients [44].

#### 2.1.7. The Effect of MSCs and MSC-Exos on Epigenetic Modifications in SLE

The relationship between MSCs and epigenetic modifications is one of mutual influence and regulation. Epigenetic changes, including DNA methylation, histone modifications, and miRNA regulation, play a crucial role in MSC differentiation [45]. MSC-exos can modulate inflammation by regulating DNA methylation levels, which in turn affect the expression of genes involved in epigenetic modifications, contributing to the improvement of SLE [46].

#### 2.1.8. Clinical Trials in SLE of MSCs

Promising clinical results in SLE mice have led researchers to treat four cyclophosphamide (CTX)/glucocorticoid treatment-refractory SLE patients using allogenic BMSC transplantation. All treated patients showed stable 12–18 months of disease remission, satisfactory control of disease activity, and decreased albuminuria and serum autoimmune antibodies. This is the first evidence that allogeneic BMSC transplantation is safe and effective for treating refractory SLE [47]. Over the past 5 years, few clinical trials on stem cell transplantation for SLE have been reported. In 2022, a systematic review and meta-analysis of randomized controlled trials of MSC transplantation for autoimmune disease showed that in a multicenter clinical study recruiting 40 SLE patients from four centers for allograft hU-MSC transplantation, each patient received two intravenous MSC transfusions 1 week apart [48]. After 12 months of follow-up, six nontransplant-related adverse events were identified. The overall survival rate posttransplantation was 92.5%, and the clinical response rate was 60%, with no serious adverse reactions reported [49]. In a long-term follow-up of up to 6 years in nine patients with refractory SLE, no serious adverse events were observed after two transfusions of allogeneic hU-MSCs, except for one patient who developed dizziness and heat sensation 5 minutes postinfusion, which quickly subsided. After 6 years, peripheral white blood cell, red blood cell, and platelet (PLT) counts remained unchanged, and liver function was normal. Tumor markers (AFP, CEA, CA125, CA199) were not elevated. Overall, allogeneic hU-MSCs have a good safety profile in SLE patients [50].

In summary, MSCs modulate the body's immune system, reducing the autoimmune response in SLE patients and aiding in re-establishing immune tolerance. This minimizes the autoimmune response and alleviates organ and tissue damage. However, further research is essential to determine whether the immunosuppressive properties of MSCs could potentially lead to infections or tumors when treating SLE.

### 2.2. MSCs and RA

RA is a chronic autoimmune disease characterized by joint pain and swelling, which can progress to joint stiffness and deformity over time. In severe cases, RA can result in loss of function and disability. The exact cause of RA is believed to involve immune, genetic, and environmental factors. Research has identified that increased levels of cytokines, triggered by both innate and adaptive immune responses, are closely linked to RA development [51]. An imbalance between Th17 cells and Tregs can trigger the onset of RA. Additionally, patients with RA often exhibit elevated levels of TNF-*α*, IL-1*β*, IL-6, and IL-17, which are crucial in promoting inflammation, attracting and sustaining inflammatory immune cells, and driving tissue damage [52]. As a result, MSCs can help treat RA by restoring immune cell balance and modulating inflammatory factors in the body [53].

#### 2.2.1. Regulation of T Cells by MSCs in RA

Research has shown that MSCs can suppress the activity of proinflammatory Th17 cells while promoting the development of Tregs. This effect is mediated by the secretion of immunosuppressive cytokines, including IL-6, IL-8, and TGF-*β* [54], which reduce Th17 cell proliferation and inhibit the release of inflammatory factors such as IL-17. The reduction in IL-17 serves as a negative feedback mechanism that further suppresses Th17 cell activation and expansion.

#### 2.2.2. Regulation of B Cells by MSCs in RA

Plasma cells, which produce antibodies, form when B2 cells differentiate during adaptive immunity. These cells are closely linked to RA disease activity [55]. RA patients often have abnormally high numbers of autoreactive B cells. MSCs can interact directly with B cells to reduce plasma cell formation. Additionally, MSCs can inhibit B lymphocyte proliferation and maturation through the COX-2 pathway, reducing the inflammatory response associated with these cells [56]. Studies show that MSCs can inhibit B cell growth and activation by reducing the expression of CD69 and CD86, and by inhibiting plasma cell differentiation and immunoglobulin IgG production [57]. MSCs also suppress effector B cell activation by reducing IL-4 secretion by B cells. Moreover, MSCs induce the conversion of B cells into regulatory B cells that produce IL-10, further enhancing MSCs' inhibitory effect on B cell activation [58].

#### 2.2.3. Regulation of Macrophages by MSCs in RA

In an inflammatory environment, MSCs can convert macrophages from the M1 (proinflammatory) phenotype to the M2 (anti-inflammatory) phenotype through the actions of enzymes and molecules such as diamine 2,3 dioxygenase (IDO) [59], CC motif ligand 18, and PGE2. Additionally, hUC-MSCs can modulate macrophage immune functions by reducing serum levels of proinflammatory factors and chemokines while increasing the anti-inflammatory cytokine IL-10, thus promoting the repair of inflammation-induced tissue damage [60]. Furthermore, hUC-MSCs can attenuate excessive inflammatory responses induced by macrophages through the inhibition of lipopolysaccharide (LPS) activity. Pretreatment with hUC-MSCs results in enhanced macrophage-mediated anti-inflammatory effects [61]. Moreover, MSC-exos inhibit M1 macrophage pyroptosis via the NLRP3/Caspase-11/GSDMD-N pathway [62]. Collectively, MSCs can delay the progression of RA inflammation and restore immune balance [63].

#### 2.2.4. Regulation of Inflammatory Cytokine Secretion by MSCs in RA

Recent animal studies have shown that MSCs can effectively inhibit the excessive expression of proinflammatory cytokines in RA patients. A study by Tian et al. [64] randomly selected 49 RA patients and measured their TNF-*α*, IL-1*β*, IL-6, and IL-8 levels triggered by MSC secretions using enzyme-linked immunosorbent assay (ELISA) and real-time fluorescence-based quantitative polymerase chain reaction (qPCR). It was found that MSC secretions significantly reduced these cytokines' expression in the patients' sera. Additionally, microvesicles (MVs) from human umbilical cord MSCs MVs (hUC-MSCs-MVs) have been found to effectively alleviate RA symptoms. Treatment of DBA/1J mice with collagen-induced arthritis (CIA) with these MSCs-MVs showed reduced expression of inflammatory factors such as IL-1*β*, IL-6, and TNF-*α* compared to the control group. Histological analysis also showed reduced inflammation in the mice's ankle joints [65].

#### 2.2.5. MSCs Inhibit Osteoclast Function in RA Patients

When activated or overactive, osteoclasts can cause excessive bone loss. However, MSCs derived from fat tissue can counteract this by inhibiting osteoclast function. This is achieved through the release of specific proinflammatory factors and chemokines, the promotion of osteoprotegerin production, and the blockade of osteoclast precursor formation via a CD200/CD200R-dependent mechanism [53]. Additionally, TGF-*β* secreted by MSCs has been found to modulate components of the extracellular matrix, either directly or indirectly, aiding in the repair of damaged joint tissues [66]. A study demonstrated that injecting BMSCs into a mouse model of spontaneous RA significantly reduced symptoms, improved femoral structures, and decreased bone loss associated with RA [67].

#### 2.2.6. MSC-Exos Regulate RA by Releasing miRNA

Matrix metalloproteinase 14 (MMP14) and vascular endothelial growth factor (VEGF) are key targets for RA treatment. Chen et al. [68] demonstrated that miR-150-5p, enriched in bone marrow-derived MSC-exosomes (BMSC-exos), directly binds to and suppresses the expression of MMP14 and VEGF. This suppression leads to a decrease in proinflammatory cytokines such as IL-1*β*, TNF-*α*, and TGF-*β*, inhibiting the proliferation and migration of fibroblast-like synoviocytes (FLS), and thereby alleviating RA-associated inflammation. Moreover, it has been reported that miR-320a from BMSC-exos can inhibit the activation, migration, and invasion of FLS in RA by targeting CXC chemokine ligand 9 (CXCL9) [69]. Additionally, another study found that incorporating miRNA-124a into MSC-exos inhibited the migration of MH7A cells (a fibroblast-like synovial cell line that mimics RA) and promoted cell apoptosis. Thus, MSC-exos can effectively control synovial inflammation, prevent cartilage erosion, and ultimately reduce the progression of RA [70].

#### 2.2.7. Clinical Trials in RA of MSCs

The immunomodulatory effects of hU-MSCs have been extensively studied in RA through in vitro and preclinical studies. Results from a human trial examining the outcomes of a single hU-MSC infusion in RA patients showed that those with moderate disease activity experienced increased cell counts without dose-limiting adverse events. During and after treatment, no subjects developed infusion reactions, serious adverse reactions, or significant abnormalities in serum chemistry or hematological characteristics, indicating no significant toxicity within 4 weeks after infusion [71]. Additionally, an Iranian clinical trial involving 9 female patients treated with a single infusion of autologous BMSCs showed significant improvement in clinical symptoms for refractory RA patients, with no complications or adverse events observed during or after MSC infusion [72]. The study later administered autologous BMSCs intravenously to 13 patients with refractory RA and followed them for 12 months postintervention to assess immunological composition. Data indicated that BMSCs significantly modulated the immune system of refractory RA patients, notably increasing protein levels of key Treg cytokines such as IL-10 and TGF-*β* [73]. In a Phase I/II nonrandomized, open-label study, 15 RA patients received a single intravenous infusion of autologous ADMSCs and were followed up at 4, 12, 26, and 52 weeks. Efficacy was measured using the American College of Rheumatology (ACR66/68 score) Swollen and Tender Joint Count (S/TJC) criteria, and serum levels of TNF-*α*, IL-6, CRP, and ESR. By the end of the 52-week follow-up, both swollen and tender joint scores showed clinically significant improvement, CRP levels showed a slight improvement, and IL-6, TNF-*α*, and ESR levels remained unchanged. No safety risks or significant adverse events were found, indicating that a single infusion of autologous ADMSCs is safe and effective in improving joint symptoms in RA patients [74].

In summary, MSCs have the potential to ameliorate RA symptoms by suppressing the functions of proinflammatory cells such as T cells, B lymphocytes, DCs, NK cells, macrophages, and osteoclasts, while enhancing those of Tregs. However, most current research on MSCs is centered on animal testing, and the therapeutic effectiveness of MSCs in the clinical treatment of RA requires further investigation. Despite this, MSCs are poised to play a significant role in the future treatment of RA and improve the quality of life for RA patients.

### 2.3. MSCs and T1DM

Diabetes mellitus (DM), commonly known as diabetes, is a prevalent disease that poses significant harm to the human body, second only to cancer. It is classified into four types: T1DM, Type 2 diabetes (T2DM), gestational diabetes, and other forms such as those caused by cell genetic defects, inherited insulin resistance, pancreatic disease, and hormone imbalances [75]. Among these, T1DM is an autoimmune disease where the body's T cells attack and destroy pancreatic *β* cells, leading to inflammation and inadequate insulin secretion [76]. The exact causes of T1DM are not fully understood, but it is believed to be associated with genetic and environmental factors, insulin resistance, obesity, and the PD-1/PD-L pathway [77]. Current research suggests that in addition to traditional medications, stem cell transplantation shows great promise in treating T1DM.

#### 2.3.1. Regulation of T Cells by MSCs in T1DM

The T cells that primarily contribute to the destruction of *β* cells in T1DM are Th1 cells. Th1 cells stimulate CD8^+^ T cells and macrophages by producing cytokines like IL-2, thereby enhancing the inflammatory response against islet *β* cells [78]. Research indicates that the proinflammatory factor TNF-*α* can induce the transformation of Th1 and Th17 cells into Tregs through the anti-inflammatory factors produced by MSCs, thereby inducing immunosuppression. During this process, the expression of PD-L1 also increases [79]. A study by Guo [80] found that co-culturing BMSCs with spleen cells from T1DM mice led to reduced Th1 cells and increased Tregs. Furthermore, PD-L1 expression in BMSCs and PD-1 in the spleen cells of T1DM mice increased at both the RNA and protein levels [80]. This suggests that MSCs can regulate T cell subsets, with the PD-L1/PD-1 interaction playing a significant role in this regulation.

#### 2.3.2. Mitochondrial Transfer by MSCs in T1DM

Recent studies have confirmed that activated CD8^+^ T cells can absorb mitochondria from adjacent MSCs, leading to the downregulation of T-bet expression, which promotes immunosuppression and inhibits IFN-*γ* secretion by CD8^+^ T cells [81]. Therefore, MSCs not only inhibit the proliferation and differentiation of CD8^+^ T cells by regulating transcription factors, but also suppress IFN-*γ* production, further promoting immunosuppression.

#### 2.3.3. Regulation of B Cells, DCs, and NK Cells by MSCs in T1DM

MSCs significantly reduce the expression of chemokine receptors such as CXCR4, CXCR5, and CCR7 on B cells [82]. Additionally, by altering the cytokine secretion profile of DCs, BMSCs can induce a more anti-inflammatory or tolerant phenotype in DCs. Contact between BMSCs and NK cells reduces the expression of activating receptors, such as NKG2D and CD69, on NK cell surfaces. Moreover, BMSCs secrete bioactive substances, including IDO, PGE2, and TGF-*β*1, which inhibit the cytotoxic activity of NK cells [83].

#### 2.3.4. Regulation of Macrophages by MSCs in T1DM

Macrophages play a critical role in the healing of diabetic ulcers [84]. As mentioned earlier, macrophages are classified into M1 and M2 subtypes [85]. M2 macrophages are effective at clearing tissue debris and apoptotic cells, promoting wound healing, and alleviating diabetic symptoms [86]. Recent studies suggest that hUC-MSCs can convert M1 macrophages into M2 macrophages, thereby assisting in diabetic wound healing, reducing inflammation of islet *β* cells, and protecting these cells [87].

#### 2.3.5. Regulation of Insulin Secretion by MSCs in Patients With T1DM

MSCs have the potential to transform into insulin-producing cells (IPCs). Pancreatic *β* cells, which constitute ~70% of all islet cells, are primarily responsible for secreting insulin and regulating blood sugar levels. Recent research suggests that inducing the conversion of umbilical cord-MSCs (UC-MSCs) into IPCs could be a promising strategy for treating diabetes. This differentiation can be triggered through several methods, including multistep induction, genetic engineering, and co-culture [88]. There are two main pathways by which MSCs transform into IPCs. The first pathway involves nestin-positive cells, where stem cells are stimulated to become nestin-positive before being reinduced to transform into IPCs. The second pathway involves the pancreas/duodenum homeobox protein 1 (PDX1)-associated pathway, where stem cells are first induced to become mature endoderm cells expressing PDX1, followed by their transformation into IPCs [89].

In a study by Yuan et al. [90], specific markers of in vitro-cultured UC-MSCs were identified using flow cytometry. They induced hUC-MSCs to transform into islet-like cells through a step-by-step induction process involving *β*-mercaptoethanol and high glucose. The expression of PDX1 and insulin in the cells was confirmed by RT-PCR and positive dithizone staining. These findings suggest that hUC-MSCs can be induced to become islet cells with insulin-secreting functionality. Earlier studies successfully transformed hUC-MSCs into insulin-secreting cells in the laboratory using serum-free culture conditions. These differentiated cells exhibited typical features of insulin-secreting cells [88]. In a study led by Shan et al. [91], hUC-MSCs were induced to different stages and then transplanted into diabetic mice to determine the ideal induction period for treating diabetic rats. The results showed reduced glucose levels in the rat blood, with the most effective treatment apparent after 28 days of in vitro induction.

Additionally, the therapeutic effect of MSCs in T1DM has been observed through the reduction of islet inflammation and the increase in plasma and islet insulin content, which helps alleviate hyperglycemia. However, this effect was diminished by the gut microbiota [92]. This may provide a potential explanation for why some patients do not respond to MSC therapy.

#### 2.3.6. MSCs and MSC Derivatives Regulate T1DM by Releasing miRNA

Diabetic nephropathy (DN) and diabetic cardiomyopathy (DCM) are severe complications of DM. Previous studies have shown that miR-146a-5p is significantly downregulated and negatively correlated with renal injury in DN rats. Furthermore, miR-146a-5p derived from hUC-MSCs can promote M2 macrophage polarization by inhibiting the tumor necrosis factor receptor-associated factor-6 (TRAF6)/signal transducer and activator of transcription 1 (STAT1) signaling pathway [93]. Additionally, intravenous injection of hUC-MSCs ameliorated key functional and structural features of DCM in male mice with both short-term and long-term diabetes. Mechanistically, these effects were associated with the restoration of intra-myocardial expression of miRNA-133a and its target mRNA COL1A1, as well as the suppression of systemic and localized inflammatory mediators [94]. Refractory diabetic wounds are a common problem in diabetic patients, often linked to epidermis-specific macroautophagy/autophagy impairment. One study demonstrated that hypoxic BMSC-exos (hyBMSC-exos) mediated the transfer of miR-4645-5p, which inactivated mitogen-activated protein kinase-activated protein kinase 2 (MAPKAPK2)-induced AKT-mTORC1 signaling in keratinocytes. This activation of keratinocyte autophagy, proliferation, and migration resulted in diabetic wound healing in mice [95]. Additionally, research has revealed that PLMSCs-MVs can induce PLMSCs to differentiate into pancreatic *β*-like cells, significantly reducing both random and fasting blood glucose levels. Importantly, this induction effect of MVs is achieved through the miR-181a-5p and miR-150-5p they carry [96].

#### 2.3.7. Clinical Trial in T1DM of MSCs

A pilot study included 13 patients (eight patients in Group 1; five patients in Group 2) with recently developed T1DM. Group 1 received ADMSCs (1 × 10^6^ cells/kg) and cholecalciferol (2000 IU/day) for 3 months, while Group 2 was treated with standard insulin. After 3 months, no serious adverse events were observed. Immediate transient adverse events in Group 1 included transient headaches (*n* = 8), mild local infusion reactions (*n* = 7), tachycardia (*n* = 4), and abdominal cramps (*n* = 1). There were no significant changes in C-peptide levels in both groups over time. Patients who received ADMSCs and cholecalciferol had better blood sugar control and lower insulin requirements compared to the standard treatment group. Although the study sample was small and the follow-up period was short, this pilot study is an important early assessment of ADMSC and vitamin D supplementation as potential combination therapies for T1DM [97].

Additionally, a preliminary report from a Phase I clinical trial in 2021 involved intravenous implantation of placenta-derived MSCs (PLMSCs) in four adolescents with T1DM. Assessments were conducted weekly for the first month, monthly for 6 months, and then every 3 months up to 1 year. The results showed no serious adverse events, including anaphylactic shock or hypersensitivity reactions, during the 1-year follow-up. Two patients experienced partial remission and episodes of hypoglycemia 1 month after transplantation. This Phase I clinical trial demonstrated the short-term safety of PLMSCs transplantation in adolescents with T1DM. However, more research is needed to demonstrate the long-term safety and efficacy of this treatment [98]. A clinical trial of BMSCs transplantation in newly diagnosed T1DM patients also demonstrated the safety of MSCs transplantation. Twenty-one patients were recruited and randomly assigned to receive MSCs or a placebo. Each patient received two doses of MSCs and was followed for at least 1 year after transplantation. The results showed that MSCs transplantation significantly reduced the number of hypoglycemia episodes, improved glycated hemoglobin (HbA1c), shifted the serum cytokine pattern from proinflammatory to anti-inflammatory, increased the number of regulatory T cells in peripheral blood, and improved quality of life [99].

In summary, cell therapies involving MSCs hold great promise and have been safely used in treating T1DM. Most importantly, in addition to regulating immune cells, MSCs can also be converted into IPCs to make up for insufficient insulin secretion in T1DM patients, thereby reducing blood sugar. However, the possibility of long-term complications cannot be ruled out. Therefore, before such therapies can be widely applied in clinical practice, numerous challenges related to safety need to be addressed.

### 2.4. MSCs and MS

MS is a common autoimmune disease that primarily affects the nervous system, often resulting in neurological dysfunction in young adults [100]. It is primarily caused by multifocal inflammation within the central nervous system (CNS) and is characterized by inflammatory damage, demyelination, and axonal loss [101]. The primary treatment for MS currently involves the use of immunosuppressants. However, long-term use of these drugs can lead to severe side effects, such as infections, cardiotoxicity, and depression [102, 103]. Therefore, there is a pressing need for new, effective treatments with fewer adverse effects [7]. Cellular immunotherapy, particularly the use of MSCs, has garnered significant attention as an emerging treatment strategy. Experimental autoimmune encephalomyelitis (EAE) shares similar pathological characteristics and clinical symptoms with MS, making it a commonly used model for research.

#### 2.4.1. Regulation of T Cells and B Cells by MSCs in MS

Zhai [104] overexpressed the BMSC secretion factor in EAE model mice and observed that B lymphocytes influenced T cell subgroups. Specifically, the secretion factor inhibited T cell differentiation into proinflammatory T cells in EAE, significantly decreasing the proportion of proinflammatory Th1 and Th17 cells while significantly increasing the proportion of Tregs. This inhibition blocked the progression of EAE and delayed its development. Liu[100] used hUC-MSCs in an EAE model in cynomolgus monkeys and observed significantly increased numbers of Tregs in the treatment group, significant decreases in the proportions of Th1 and Th17 cells, and a significant increase in the expression level of soluble cell differentiation antigen 40 ligand (SCD40L) in the peripheral blood. Zhou et al. [102] transfected ADMSCs with the sphingosine kinase 1 (SPK1) gene in a mouse EAE model. They observed an increased Th17/Treg ratio in the EAE mice, which was significantly reduced after ADMSC treatment, especially after ADMSC-SPK1 transplantation. Zhai and colleagues treated EAE model mice with the BMSC secretion factor and investigated its addition to co-cultured T and B cells in vitro. The BMSC secretion factor reduced the proportion of proinflammatory B cells and increased the proportion of Bregs [104], suggesting that it can inhibit and regulate inflammation.

#### 2.4.2. Modulation of Cytokines by MSCs in MS

Xue and An [105] treated EAE model mice with human neural stem cells (hNSCs), resulting in reduced infiltration of inflammatory cells in the mouse brain tissue and decreased levels of the proinflammatory factors IL-1*β* and IL-6 [9]. Conversely, there was an increase in the levels of the anti-inflammatory factor IL-10, indicating that hNSC treatment could alleviate EAE.

#### 2.4.3. MSCs Can Reduce the Generation of Demyelinating Lesions

MSCs have been found to suppress astrocyte activation, reduce the formation of demyelinating lesions, and alleviate symptoms of EAE [100]. In a completed clinical trial (No. NCT02034188), 20 subjects aged 18–55 from different countries received seven intravenous infusions of UC-MSCs (one/day) over 7 days. The treatment significantly improved MS clinical symptoms. Some participants reported mild side effects, such as headache and fatigue, during the follow-up period, but no other adverse effects were observed. MRI scans revealed no further disease progression in 83.3% of the participants a year after treatment. Although the lesions in the brain and spinal cord did not diminish or fade, clinical symptoms were significantly alleviated [10, 11, 106].

Various types of MSCs have been used in the treatment of EAE, resulting in significant delays in disease progression [101]. However, the exact mechanisms by which MSCs exert their influence have not been fully elucidated. One possibility is that MSCs inhibit certain functions of B cells, which in turn affects the proportions and functions of T cells [104]. This modulation of T cells reduces the secretion of T-cell-related cytokines, resulting in the alleviation of inflammation. Another potential mechanism is the direct impact of MSCs on the balance between Th1 and Th17 cells, increasing the proportion of Tregs and enhancing immune regulation. A study by Liu utilized a nonhuman primate model to evaluate the therapeutic potential of hUC-MSCs in MS. The study found that hUC-MSCs did not promote proliferation in the lung adenocarcinoma cell line SPC-A-1 or tumor metastasis in nude mice and crab-eating monkeys [100].

#### 2.4.4. MSC Derivatives Regulate MS by Releasing miRNA

Recent studies have demonstrated the regulatory effects of human umbilical MSC-derived exosomes (hU-MSC-exos) on oligodendrocyte cells, which are involved in myelinogenesis in MS. This effect is primarily mediated through the transfer of miR-23a-3p via these exosomes. Functional investigations revealed that hU-MSC-exos activate the PI3k/Akt pathway and regulate Tbr1/Wnt signaling molecules through miR-23a-3p, promoting oligodendrocyte differentiation and enhancing the expression of myelin-related proteins [107]. Additionally, another study involved injecting engineered EVs containing miR-181a-5p into mice with EAE, an established animal model of MS. The results showed that these EVs mitigated spinal cord injury and demyelination in EAE mice. Mechanistically, MSC-EVs containing miR-181a-5p inhibited microglial inflammation and pyroptosis via the ubiquitin-specific protease 15 (USP15)-mediated RelA/NEK7 axis, thereby alleviating the clinical symptoms of EAE [108].

#### 2.4.5. Clinical Trials of MSCs in MS

A recent clinical study evaluated the safety and efficacy of injecting different doses of umbilical cord-derived MSCs (UC-MSCs) into MS patients. The study included two groups of patients: Group A received two intrathecal doses of UC-MSCs, while Group B received a single dose. After 3 months, no serious adverse events were reported in either group. 6 months posttreatment, both groups showed significant improvements on the general disability scale, with Group A exhibiting more parameters of improvement compared to Group B [109]. Additionally, MSC-neural progenitors (MSC-NPs), a bone marrow-derived ex vivo manipulated cell product, have shown therapeutic potential in MS. A Phase II, randomized, placebo-controlled clinical trial found that MSC-NP treatment had significant efficacy in patients with progressive MS compared to the control group. Indirect evidence of neuroprotective effects was also observed in brain MRI, specifically in cortical gray matter volume changes [110].

In summary, MSC transplantation can inhibit inflammatory responses and alleviate demyelinating lesions in the CNS, making it a potential treatment for MS. This method has few side effects, can effectively alleviate patient suffering, delay disease progression, and improve clinical symptoms. Therefore, MSC transplantation may become a new option for treating MS.

### 2.5. MSCs and ITP

ITP is an AID characterized by immune-mediated PLT destruction, disrupted immune tolerance, and severe bleeding in the skin, mucous membranes, and internal organs. Its complex pathogenesis involves both humoral and cellular immune regulation and is influenced by factors such as infection, genetics, pregnancy, and drug-induced oxidative stress [111]. The primary treatments for ITP are hormone therapy and intravenous immunoglobulin administration. However, these treatments are not always effective and may lead to infection or recurrence after an initially satisfactory response. Patients with recurrent ITP often undergo splenectomy, a treatment option with significant risks [112]. Emerging treatments, such as stem cell transplantation, are gaining attention for their potential to reduce patient trauma.

#### 2.5.1. Regulation of T Cells by MSCs in ITP Patients

Xiao and Zhang [113] administered AMSCs to ITP model mice and found that AMSC injection reduced T-bet levels while increasing GATA-3 expression, thus restoring the Th1/Th2 cytokine balance. Additionally, IFN-*γ* and IL-2 levels decreased, while IL-4 and IL-10 levels increased. However, Pérez-Simón et al. [114] reported that the inhibitory effect of BMSCs on T lymphocyte proliferation was significantly weaker in adult ITP patients with refractory splenectomy compared to normal BMSCs. Similarly, Wu et al. [115] found no significant differences in the ability of BMSCs from children with acute ITP to inhibit PBMC proliferation compared to BMSCs from normal children. This suggests that BMSCs may differ in their ability to regulate lymphocyte proliferation in chronic refractory and acute ITP. Furthermore, Zhang et al. [116] found that senescent and apoptotic MSCs inhibited T cell proliferation in ITP while reducing Treg induction.

#### 2.5.2. MSC Derivatives Regulate ITP by Releasing miRNA

Studies have discovered that miR-199a-5p levels are lower in patients with ITP but increase following treatment, suggesting a potential role for miR-199a-5p in the condition [117]. Another study demonstrated that in ITP mice with low miR-199a-5p expression, exosomes from ADMSCs containing miR-199a-5p inhibited Th17 cell differentiation by down-regulating the expression of signal transducer and activator of transcription 3 (STAT3). This intervention effectively delayed the progression of ITP in experimental mouse models. Furthermore, the administration of miR-199a-5p-enriched exosomes significantly increased PLT counts in ITP mice [118]. Additionally, miR-146a-5p is highly expressed in BMSC-exos. Research has shown that BMSC-derived exosomal miR-146a-5p can regulate the Th17/Treg imbalance in ITP by inhibiting the expression of interleukin-1 receptor-associated kinase 1 (IRAK1) [119].

#### 2.5.3. Clinical Trials in ITP of MSCs

The above studies suggest that MSCs can alter T cell populations and regulate transcription factor expression through gene regulation, adjusting cytokine proportions and even restoring PLT levels. According to a study by Wang et al. [120], complete remission was observed in four ITP patients at varying times after injection with hUC-MSCs. Three of these patients underwent secondary transplantation with the same dosage. No serious adverse events were reported during or after transplantation, and the use of immunosuppressants was unnecessary. These findings suggest that MSCs are a safe treatment for ITP. However, additional clinical trials are required to confirm the effectiveness of MSCs in treating different forms of ITP.

In summary, MSCs can regulate the Th17/Treg ratio, inhibit DCs differentiation, and induce mDCs to become regDCs. In addition, MSCs can increase PLT levels and reduce purpura by regulating cytokines. And MSC-exos can regulate the Th17/Treg ratio and increase PLTs by releasing miRNA. In clinical trials, MSCs have shown generally good therapeutic outcomes, with only a small number of patients experiencing serious adverse events.

## 3. Summary and Outlook

### 3.1. Treatment Mechanism of MSCs

**Figure 1:**
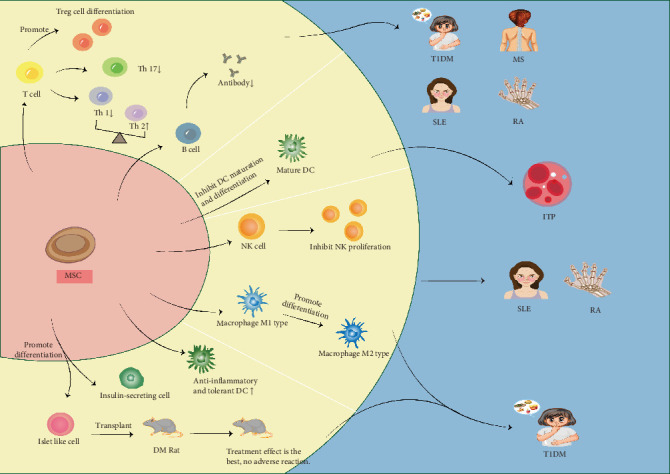
An illustration of the mechanisms underlying the role of mesenchymal stem cell (MSC) in treating systemic lupus erythematosus (SLE), rheumatoid arthritis (RA), Type 1 diabetes (T1DM), multiple sclerosis (MS), and immune thrombocytopenia (ITP). MSC play regulatory roles in different autoimmune diseases (AIDs) by inhibiting or altering the phenotypes of active cells. For example, in the figure, MSC act on T cell, promoting T cell differentiation into treg cell and Th 2, and inhibit the translation of T cell into Th 17 and Th 1. This regulates the proportions of the various immune cells together with increasing the proportion of anti-inflammatory cells, thus reducing disease-associated inflammation.

Although the comprehensive mechanisms by which MSCs contribute to the treatment of various AIDs are still being investigated, the general mechanisms are summarized as follows (see Figure 1, Tables 1 and 2):

1. MSCs inhibit T cell proliferation and reduce the proportion of proinflammatory T cells.
2. MSCs act on B cells by inhibiting their differentiation and reducing the production of autoantibodies.
3. MSCs induce macrophage polarization from the M1 to the M2 phenotype.
4. MSCs inhibit the activity of NK cells and DCs, thereby reducing the inflammatory response.

### 3.2. The Challenges of MSCs in the Treatment of AIDs

MSCs, as an emerging therapy, are currently effective in treating AIDs and can address some issues in clinical AIDs treatment. However, several unresolved problems remain with MSC transplantation, such as donor heterogeneity, cryopreservation, and safety. First, many studies on MSC transplantation therapy are limited to in vitro or animal model experiments, and no large-scale human clinical trials have been conducted. Due to differences between humans and animals, it is challenging to accurately assess the efficacy of MSCs in humans. Second, in some clinical experiments, MSCs have shown issues with short in vivo survival times, allowing AIDs recurrence. Although secondary MSC treatment is still effective, this may increase the economic burden on patients with AIDs. Additionally, tumorigenicity is a potential problem associated with MSC transplantation. The multidirectional and irregular differentiation of cells can disrupt the intracellular environment, leading to apoptosis and teratoma formation. Therefore, future studies can further explore the possible mechanisms of MSC-induced tumorigenesis and work to mitigate these risks.

Moreover, despite numerous studies on the therapeutic potential of MSCs, the translation of these findings into clinical applications has been slower than expected. Several key factors contribute to this perceived delay. First, the biological systems in humans are more complicated than controlled laboratory settings. Understanding and replicating these complexities presents significant challenges in translating benchside discoveries to bedside treatments. The use of preclinical testing may not accurately predict human responses to MSC therapies due to subtle differences in physiology and immune responses between species, which can limit the translatability of preclinical findings. Additionally, MSCs display significant diversity influenced by factors such as donor variability, tissue source, and isolation methods. This diversity can result in inconsistencies in therapeutic outcomes, impeding the standardization and regulatory approval of MSC-based therapies. Moreover, the regulatory frameworks governing the development and approval of MSC-based therapies vary globally and are often evolving. Navigating these regulatory hurdles, such as demonstrating safety, imposes time and resource burdens on clinical trials. Considering these challenges, it is clear that there are complex obstacles to translating MSC research into clinical practice, including interdisciplinary collaboration, stringent standardization, and continual refinement of experimental and regulatory strategies. Researchers need to overcome these challenges to accelerate the translation of MSC research into impactful clinical applications.

### 3.3. Outlook

In summary, MSCs mediate therapeutic effects on AIDs primarily by regulating the expression, differentiation, and migration of immune cells (e.g., T cells, B cells, macrophages, NK cells, and DCs) and nonimmune cells (e.g., oligodendrocytes, FLS). In contrast, in nonAIDs, MSCs provide therapeutic benefits mainly through their differentiation potential, such as differentiating into dopaminergic neurons under hypoxic conditions for Parkinson's disease treatment or transdifferentiating into cardiomyocytes and endothelial cells to repair damaged myocardial tissue in both acute and chronic cardiac injuries.

The application of MSCs in treating AIDs shows significant potential. However, further research is necessary to fully elucidate the mechanisms underlying their therapeutic effects and to explore potential risks and limitations. Future research should focus on enhancing the isolation and characterization of MSCs to identify the optimal cell types with the most effective immunomodulatory properties, thereby improving their therapeutic potential in various AIDs. Given the complexity and high costs associated with exosome extraction and purification, developing more efficient and economical techniques in this area is crucial. Additionally, improving the labeling and tracking techniques for exosomes can better monitor their distribution and metabolic processes in vivo, offering valuable guidance for clinical applications. Initiating more clinical trials to investigate the use of MSCs as primary or adjunct therapies for rare AIDs with limited treatment options could expand our understanding of MSC efficacy across a broader spectrum of diseases. Therefore, multicenter, large-sample clinical trials are recommended to evaluate the safety and efficacy of MSC therapy. Clinical trials should include rigorous patient screening criteria, standardized cell preparation and infusion procedures, and long-term follow-up. Through these clinical trials, we can provide a scientific basis for the application of MSCs in AIDs and provide data support for the development of future treatment guidelines.

## Figures and Tables

**Table 1:** The regulatory roles of mesenchymal stem cells (MSCs) in autoimmune diseases (AIDs).

Diseases	MSC types	Targets	Mechanisms	References
SLE	BMSCs	T cells	Elevate treg cells differentiation	[27]
	BMSCs	CD8^+^ T cells	Inhibit CTL toxicity	[28]
	BMSCs	T cells	Inhibit the translation of Th0 into Th17 and Th1, promote Th0 translating into Th2	[29]
	BMSCs	B cells	PDL-1 and PD-1 interact to inhibit B cells proliferation	[32–34]
	Human fetal liver MSC-exos	NK cells	Inhibit NK cells proliferation	[36, 37]
	BMSCs, hUC-MSCs	DCs	Inhibit secretion of INF-*γ*, IL-12, IL-6, TNF-*α*, and DCs function	[36, 38]
	BMSC-exos	Macrophages	Promote the translation of M1 macrophage into M2 macrophage	[39–41]

RA	BMSCs	T cells	Elevate treg cells differentiation and inhibit Th17 differentiation	[54]
	hA-MSCs, BMSCs	B cells	Inhibit B cells proliferation	[55–58]
	BMSCs	DCs	Inhibit DCs maturation	[37]
	hUC-MSCs	Macrophages	Promote the translation of M1 macrophage into M2 macrophage	[59–61]
	BMSCs	NK cells	Inhibit NK activation and proliferation	[59]
	BMSC-exos	fibroblast-like synoviocytes	Inhibited proliferation and migration of FLS	[68, 69]
	MSC-exos	MH7A cells	inhibit the migration of MH7A cells and promote cell apoptosis by adding miRNA-124a in exosomes	[70]

T1DM	BMSCs	T cells	Promote the translation of Th1 and Th17 into treg cells	[79, 80]
	BMSCs	B cells	Inhibit B cells activation and proliferation and promote bregs proliferation	[82, 83]
	BMSCs	DCs	Induce anti-inflammatory and tolerant DC	[83]
	BMSCs	NK cells	inhibit the cytotoxic actions of NK cells	[83]
	hUC-MSCs	Macrophages	Promote the translation of M1 macrophage into M2 macrophage and protect pancreatic *β* cells	[88–91]
	hUC-MSC-exos	Macrophages	Promote the translation of M1 macrophage into M2 macrophage by releasing miR-146a-5p	[93]

MS	hUC-MSCs	T cells	Elevate treg cells differentiation and inhibit Th1 and Th17 differentiation	[100, 102, 104]
	BMSC-exos	B cells	Reduce the proportion of proinflammatory B cells and increase the proportion of regulatory B cells	[104]
	hUC-MSC-exos	oligodendrocytes	Promote oligodendrocyte differentiation and enhancing the expression of myelin-related proteins	[107]

ITP	BMSCs	T cells	Elevate the ratio of Th1/Th2	[121]
	BMSCs	DC cells	Inhibit the differentiation of DC cells derived from CD34^+^ HPCs or monocytes and intervening with process of expressing the costimulator CD80/CD86 and IL-12 while intervening with differentiation of T cells	[122, 123]
	AMSC-EVs	miR-199a-5p	Inhibit Th17 differentiation and increase PLT counts	[117, 118]
	BMSC-exos	T cells	Regulate Th17/treg imbalance	[119]

**Table 2:** Summary of mesenchymal stem cell (MSC) mechanisms in autoimmune diseases (AIDs).

Target	SLE	RA	T1DM	MS	ITP
T cells	TGF-*β*1→Treg ↑/Th17↓ [27]	Treg↑/Th17↓ [54]	PD-1/PD-L1→Treg↑/Th1↓ [80]	Treg↑/Th17↓ [104]	T-bet↓/GATA-3↑ [113]
B cells	PD-L1/CCL2 [32, 33]	COX-2→Breg↓ [56]	CXCR4/CXCR5/CCR7↓ [82]	Breg↑ [104]	—
Macrophages	tSNA-21109→M2 [44]	IL-10↑/LPS↓ [60]	M1→M2 [87]	—	—
miRNA	miR-320b→MAP3K1 [15]	miR-150-5p→MMP14 [68]	miR-146a-5p→TRAF6 [93]	miR-23a–3p→PI3k/Akt [107]	miR-199a-5p→STAT3 [118]

## Data Availability

The data sharing is not applicable to this article as no datasets were generated or analyzed during the current study.
